# Simulation model of disease incidence driven by diagnostic activity

**DOI:** 10.1002/sim.8833

**Published:** 2020-11-25

**Authors:** Marcus Westerberg, Rolf Larsson, Lars Holmberg, Pär Stattin, Hans Garmo

**Affiliations:** ^1^ Department of Mathematics Uppsala University Uppsala Sweden; ^2^ Department of Surgical Sciences Uppsala University Uppsala Sweden; ^3^ Translational Oncology & Urology Research (TOUR), School of Cancer and Pharmaceutical Sciences King's College London London UK

**Keywords:** incidence, population‐based, prostate cancer, real‐life, screening, simulation model

## Abstract

It is imperative to understand the effects of early detection and treatment of chronic diseases, such as prostate cancer, regarding incidence, overtreatment and mortality. Previous simulation models have emulated clinical trials, and relied on extensive assumptions on the natural history of the disease. In addition, model parameters were typically calibrated to a variety of data sources. We propose a model designed to emulate real‐life scenarios of chronic disease using a proxy for the diagnostic activity without explicitly modeling the natural history of the disease and properties of clinical tests. Our model was applied to Swedish nation‐wide population‐based prostate cancer data, and demonstrated good performance in terms of reconstructing observed incidence and mortality. The model was used to predict the number of prostate cancer diagnoses with a high or limited diagnostic activity between 2017 and 2060. In the long term, high diagnostic activity resulted in a substantial increase in the number of men diagnosed with lower risk disease, fewer men with metastatic disease, and decreased prostate cancer mortality. The model can be used for prediction of outcome, to guide decision‐making, and to evaluate diagnostic activity in real‐life settings with respect to overdiagnosis and prostate cancer mortality.

## 1 INTRODUCTION

Some chronic diseases have a long preclinical stage that is detectable using a clinical test. In some of these diseases, life‐prolonging treatments are available for patients detected with the disease at this stage. The intensity of the current diagnostic activity affects how likely subjects in a preclinical stage are to be investigated. By combining this level of intensity with the history of diagnostic activity, one can determine how likely these subjects will be diagnosed with the disease. Under these circumstances, it is essential to have a comprehensive understanding of the causal and temporal dynamics of disease incidence, mortality, and diagnostic activity. For example, prostate‐specific antigen (PSA) testing for prostate cancer increases the incidence of low‐risk prostate cancer and decreases the incidence of metastatic prostate cancer,^1^, ^2^, ^3^, ^4^, ^5^ but is still debated as it leads to overdiagnosis and overtreatment.^6^

Often, observational data and micro‐simulations using natural history models are used as to complement randomized trials in an effort to assess the effectiveness of early diagnosis and treatment (eg, the prostate cancer screening model developed at the Fred Hutchinson Cancer Research Centre, FHCRC). The FHCRC model^7^ has recently been applied using Swedish registry data,^8^ and several instances of the Microsimulation Screening Analysis model (MISCAN) have been applied to prostate cancer data.^9^, ^10^, ^11^, ^12^ Similar models have been applied to ovarian and breast cancer data.^13^, ^14^, ^15^ These models are based on assumptions on the natural history, lead time, disease progression, disease detection, and properties of clinical tests. Typically, these models are calibrated using several data sources such as registries, clinical trials, and population‐based life tables and may cover selected populations from different countries.

We describe a model that may be used to compare current clinical practice against historical practice in a given country or to produce prognoses of incidence and mortality in real‐life situations. Because the aim is not to compare the effectiveness of a screening strategy by emulating clinical trials, there is no need for detailed modeling of the natural history of the disease and effects of the tests. Moreover, combinations of parameter estimates from clinical trials (typically with selected subjects) and of estimates and calibration targets from various populations might not apply directly to the real‐life setting in a specific country.

Therefore, our primary goal is to develop a different type of model of risk‐stratified incidence without explicit assumptions on lead time, natural history, or test properties. A key assumption in our model is that there are several experimental units, defined by, for example, region of residence and birth year, and a proxy for diagnostic activity in each experimental unit that varies between units, age groups, and time, corresponding to a quasi‐experimental design. Our secondary goal is to develop a submodel of time and cause of death after diagnosis. In addition, we show that the model is easily combined with imputation methods for handling missing data. Next, we apply the model to population‐based prostate cancer data, hereon referred to as the Proxy‐based Risk‐stratified Incidence Simulation Model – Prostate Cancer (PRISM‐PC), to explore the effects of increased diagnostic activity primarily driven by unorganized PSA testing on incidence and number of diagnoses and prostate cancer mortality in a real‐life setting.

## 2 METHODS

### 2.1 State transition model

**FIGURE 1:**
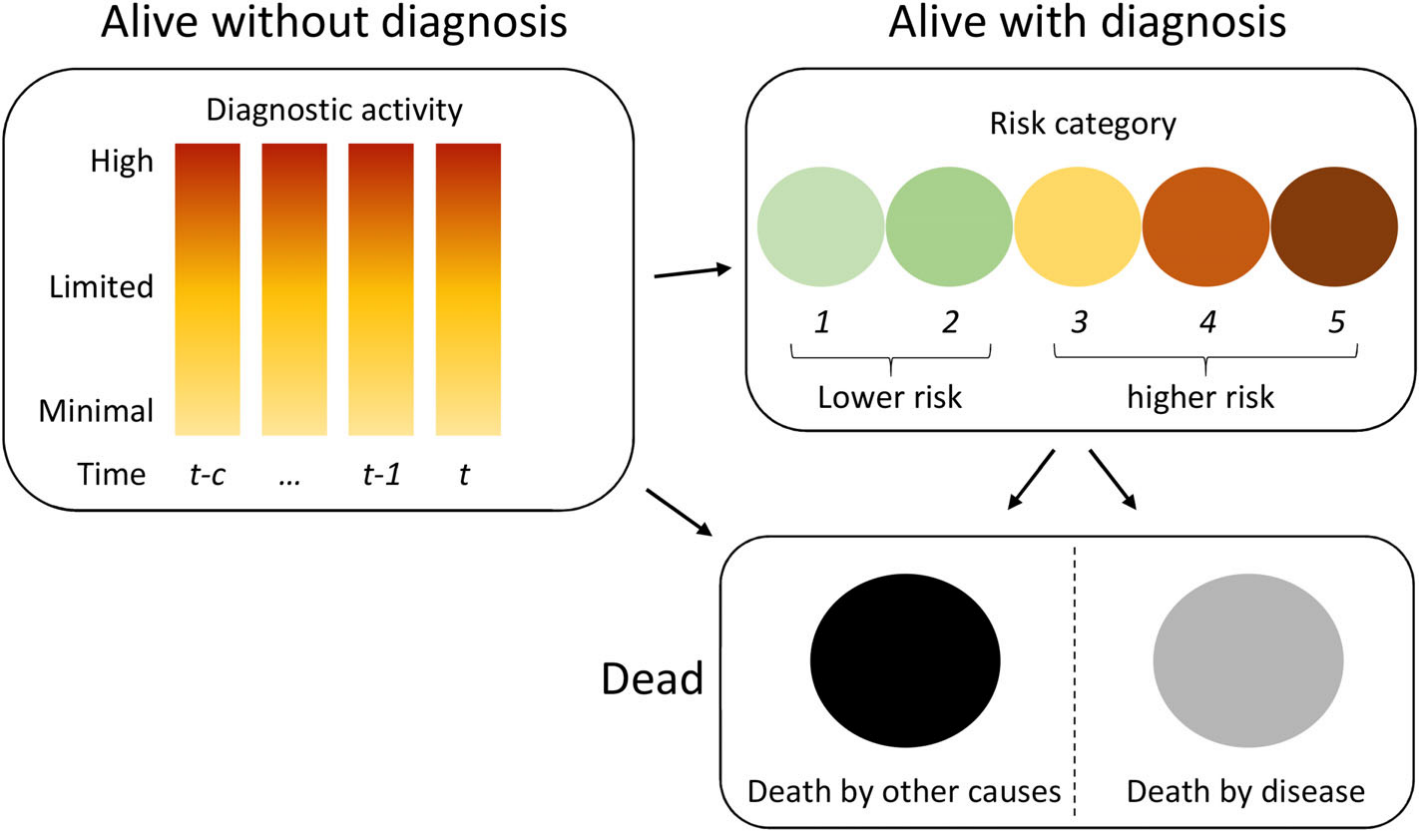
Example of the state transition model based on the history of diagnostic activity up to *c* time units back and with five risk categories [Colour figure can be viewed at wileyonlinelibrary.com]

We use a discrete time and discrete state transition model that describes risk‐stratified disease incidence based on a proxy for the level of diagnostic activity, see Figure 1. It consists of three model components: the first determines the proxy, the second determines incidence given the proxy, and the last determines mortality for those with diagnosed disease.

Before defining each model component, we present the assumptions that underlie the model. The disease is assumed to be detectable due to symptoms or by nonsymptomatic clinical testing, and the disease risk categories are assumed to be ordered in the following way. The risk categories are grouped into two subsets, lower and higher, and a typical untreated (or latent) disease progression goes from lower risk to higher risk with time since disease onset; however, we do not assume ordered transitions within each subset. For example, for the risk categories 1 to 5 (lower risk is 1 and 2 and higher risk is 3‐5), a latent disease can change stage within each subset (eg, from stage 1 to 2 or 3 to 5 or progress from a lower risk to higher risk such as changing from stage 2 to 3 or 1 to 4). We assume nothing regarding dwelling times in disease stages or transition rates between stages, but we assume the natural history is age dependent and stable over calendar time and geographical regions. Latent disease of higher risk disease is assumed to lead to symptoms and a majority of subjects with latent asymptomatic disease will have a lower risk disease. It is also assumed that a combination of current and past diagnostic activity affects current incidence of disease in each risk category for a given birth cohort and that this determines both the number of subjects at risk and their risk of being diagnosed with the disease, as indicated in Figure 1. Because data on the diagnostic activity are usually unavailable for an entire population, we use a proxy for the diagnostic activity based on the above assumptions in terms of a combination of age and incidence of lower risk disease per person time. It is also assumed that only subjects with disease can die because of the disease while all subjects can die by other causes.

### 2.2 Definitions

We here define all objects required to define the model, and these have been summarized in Table 1. Let *t*_first_ and *t*_last_ be the first and last time points where subjects have contributed with observed person time. Let *N* be the size of the study population consisting of subjects that are alive at some point after *t*_first_, and also including those entering the cohort after *t*_last_ until some set future time *t*_stop_ where the simulation ends, indexed from 1 to *N*. Let *I*_obs_ be the subset of indices corresponding to subjects who have been at risk of being diagnosed at some time point between and including *t*_first_ and *t*_last_, and let *I*_fut_ be the subset of indices corresponding to subjects who are at risk of being diagnosed between *t*_last_ and *t*_stop_. Note that *I*_obs_ and *I*_fut_ are not necessarily disjoint. Similarly, let *M*_obs_ be the subset of indices corresponding to subjects who have been at risk of death by disease, that is, have been diagnosed at some time up until *t*_last_ and have been alive at some time ≥ *t*_first_. Let *M*_fut_ be the subset of indices corresponding to subjects with a diagnosis who are at risk of dying between *t*_last_ and *t*_stop_. Let *X* be the a matrix with *N* rows and with columns that represent the covariates that define each experimental unit (eg, time of birth and region of residence). Let the vector *X*_*i*_ denote a row in *X*, and from hereon where applicable, define corresponding row vectors of matrices analogously.

**TABLE 1:** Summary of definitions

Object	Type	Details	
*t*_first_	Time point	Earliest time a subject can be at risk	
*t*_last_	Time point	Last observed time a subject can be at risk	
*t*_stop_	Time point	End of simulation	
*I*_obs_	Set of indices	Subjects that have been at risk of diagnosis	
*I*_fut_	Set of indices	Subjects that will be at risk of diagnosis	
*M*_obs_	Set of indices	Subjects that have been at risk of death by disease	
*M*_fut_	Set of indices	Subjects that will be at risk of death by disease	
			Model component
*X*	Covariates	Used to defines experimental units	All
*C*	Censoring times	E.g. time of emigration	
*E*_*p*_	Entry time or start of follow‐up	*E*_*p*, *i*_ : = *E*_1, *i*_	Proxy
*T*_*p*_	Event time	*T*_*p*, *i*_ : = *T*_1, *i*_	
*D*_*p*_	Time of end of follow‐up	Observed end of follow‐up (possibly censored)	
$\delta_{p}$	Indicator of complete follow‐up	$\delta_{p,i}:=\delta_{1,i}\times 1_{S_{1,i}\in LR}$	
$\theta_{p}$	Parameter vector		
*P*_*i*_	Proxy for individual *i*	Hazard of lower risk disease	
*E*_1_	Entry time or start of follow‐up	First time being at risk of diagnosis	Incidence
*S*_1_	Event type	Risk category at diagnosis or death	
*T*_1_	Event time	Time of diagnosis or death	
*D*_1_	Time of end of follow‐up	Observed end of follow‐up (possibly censored)	
$\delta_{1}$	Indicator of complete follow‐up	$\delta_{1,i}=0$ if censored, else 1	
$\theta_{1}$	Parameter vector		
*E*_2_	Entry time	First time at risk of death after diagnosis	Mortality
*S*_2_	Event type	Cause of death	
*T*_2_	Event time	Time of death	
*D*_2_	Time of end of follow‐up	Observed end of follow‐up (possibly censored)	
$\delta_{2}$	Indicator of complete follow‐up	$\delta_{2,i}=0$ if censored, else 1	
$\theta_{2}$	Parameter vector		

We now define the data present in each corresponding model component. The data consists of entry times (start of follow‐up), event times (time of diagnosis or death) and types (risk category at diagnosis, or cause of death), censoring times and indicators.^16^ Censoring (eg, due to migration) is assumed to be independent and noninformative.

To model incidence, let *E*_1, *i*_ denote the first time when subject *i* can contribute with person time as alive without diagnosis. Let *S*_1, *i*_ be a factor that indicates the risk category at diagnosis or death before diagnosis, and let *T*_1, *i*_ be the time of diagnosis or death before diagnosis. Let *C*_*i*_ denote the time of censoring (eg, time of emigration). Note that censoring is assumed to only occur in the observed time window, so for subjects with *C*_*i*_ > *t*_last_ then we let *C*_*i*_ = *∞*. Define $D_{1,i}:=\min(T_{1,i},C_{i})$ to be the last time of follow‐up. Denote the indicator function of the event *A* as **1**_*A*_, such that **1**_*A*_ = 1 when *A* occurs and else **1**_*A*_ = 0, and define $\delta_{1,i}:=1_{C_{i}>T_{1,i}}$ to be an indicator variable taking the value 1 if follow‐up is complete and 0 if it is censored.

Given a parameter vector $\theta_{p}$, we define $P_{i}:=P_{i}(X_{i},\theta_{p})$ as a matrix representing the proxy for diagnostic activity at each time point defined by age (rows in *P*_*i*_) during each subjects lifetime, as a function of covariates *X*_*i*_ and $\theta_{p}$. The first column of *P*_*i*_ contains elements in form of the probability of being diagnosed with lower risk disease vs staying alive $\lambda (X_{i},\theta_{p},t)$ where *t* denote each time point during the lifetime of subject *i*. Each next column indexed by *c* contain a time lagged version of $\lambda (X_{i},\theta_{p},t-c)$.

To obtain the proxy for diagnostic activity we need knowledge about $\theta_{p}$ which is obtained via a model of $\lambda (X_{i},\theta_{p},t)$. Let *LR* denote the subset of risk categories defined as *lower risk*. Let *E*_*p*, *i*_ : = *E*_1, *i*_, *T*_*p*, *i*_ : = *T*_1, *i*_, *D*_*p*, *i*_ : = *D*_1, *i*_, and $\delta_{p,i}:=\delta_{1,i}\times 1_{S_{1,i}\in LR}$, that is, subjects that are diagnosed with a nonlower risk disease or die before diagnosis are artificially censored.

To model mortality among those with a diagnosis, define the start of follow‐up as the first time when a subject can contribute with person time as alive with diagnosis $E_{2,i}:=\max(T_{1,i},t_{\text{first}})$. Let *S*_2, *i*_ denote the cause of death (eg, death by disease or death by other causes) and let *T*_2, *i*_ the time of death. Let $D_{2,i}=\min(T_{2,i},C_{i})$ be the last time of follow‐up $\delta_{2,i}=1_{D_{2,i}=T_{2,i}}$ be an indicator variable of complete follow‐up.

Note that subjects with index in $M_{\text{fut}}⋂I_{\text{fut}}$ have a simulated time of diagnosis, while subjects with index in *M*_obs_ with *D*_2, *i*_ = *C*_*i*_ = *t*_last_ have an observed time of diagnosis and are at risk of dying at the first time after *t*_last_.

### 2.3 The three model components and posterior distributions

We use pseudo observations for each subject at each time point where the subject is at risk,^16^ and specify each of the three model components (the proxy, incidence, and mortality) as multinomial logit models, with parameter vectors $\theta_{p}$, $\theta_{1}$, and $\theta_{2}$, respectively. The baseline or reference category is *event‐free* (eg, alive without diagnosis).^16^ To model complex nonlinear relationships between covariates (eg, age and history of diagnostic activity) we use tensor product‐based smooth functions of the covariates in each model component, and we use improper Gaussian priors on $\theta_{p}$, $\theta_{1}$, and $\theta_{2}$, to penalize functional wiggliness,^17^ and further assume prior independence of $\theta_{p}$, $\theta_{1}$, and $\theta_{2}$.

#### 2.3.1 The proxy for diagnostic activity

The posterior of the proxy in expressed terms of the posterior of $\theta_{p}$ given the observed data, which we express in terms of a likelihood and the prior using Bayes theorem as below

(1) $$\mathrm{Pr}(\theta_{p}|X,D_{p},\delta_{p},E_{p})\propto \mathrm{Pr}(\theta_{p})\underset{i\in I_{\text{obs}}}{\prod }\mathrm{Pr}(D_{p,i},\delta_{p,i}|X_{i},E_{p,i},\theta_{p}),$$

where ∝ means that the left‐hand side is proportional to the right‐hand side, and where we assume that $\mathrm{Pr}(\theta_{p}|X,E_{p})=\mathrm{Pr}(\theta_{p})$.

Note that $\lambda (X_{i},\theta_{p},t)$ is determined by observed data at times between *t*_first_ and *t*_last_, and at each time point before *t*_first_ and after *t*_last_ it must be specified using an assumption on the diagnostic activity in the experimental unit defined by *X*_*i*_. For *i* ∈ *I*_obs_, let *P*_obs, *i*_ be the submatrix of *P*_*i*_ containing only rows corresponding to times from the first observed time *E*_1, *i*_ to the last observed time *D*_*p*, *i*_. Let *P* be the set of all *P*_*i*_, and *P*_obs_ be the set of all *P*_obs, *i*_. In the models of incidence and mortality, the hazards only depend on elements of the proxy *P*_obs, *i*_ up until and including the corresponding time point.

#### 2.3.2 Incidence

This model component describes the transition probabilities from being *alive without diagnosis* to being *alive with diagnosis* (including risk category) or to being *dead* (death by other causes without a prior diagnosis). The posterior of the parameter given the observed data and the proxy is expressed terms of a likelihood and a prior using Bayes theorem as below

(2) $$\mathrm{Pr}(\theta_{1}|X,S_{1},D_{1},\delta_{1},E_{1},P_{\text{obs}})\propto \mathrm{Pr}(\theta_{1})\times \underset{i\in I_{\text{obs}}}{\prod }\mathrm{Pr}(D_{1,i},S_{1,i},\delta_{1,i}|X_{i},E_{1,i},P_{\text{obs},i},\theta_{1}),$$

where we assume that $\mathrm{Pr}(\theta_{1}|X,P_{\text{obs}},E_{1})=\mathrm{Pr}(\theta_{1})$.

#### 2.3.3 Mortality

This model component describes the transition probabilities from being *alive with a diagnosis* to being *dead* (including cause of death). The posterior is

(3) $$\mathrm{Pr}(\theta_{2}|X,S_{2},D_{2},\delta_{2},E_{2},S_{1},T_{1},P_{\text{obs}})\propto \mathrm{Pr}(\theta_{2})\times \underset{i\in M_{\text{obs}}}{\prod }\mathrm{Pr}(D_{2,i},S_{2,i},\delta_{2,i}|X_{i},E_{2,i},P_{\text{obs},i},S_{1},T_{1},\theta_{2}),$$

where we assume that $\mathrm{Pr}(\theta_{2}|X,P_{\text{obs}},E_{2},S_{1},T_{1})=\mathrm{Pr}(\theta_{2})$.

### 2.4 Estimation and simulation

The posterior distributions of the parameters can be approximated by maximizing the log‐likelihood functions using restricted maximum likelihood (REML) where the prior distributions effectively function as penalization terms. The approximate posterior distributions are normal distributions with means at the corresponding maximum a posterior estimates and covariance matrices equal to the estimated Bayesian posterior covariance matrices ${\hat{\sum }}_{P},{\hat{\sum }}_{1}$, and ${\hat{\sum }}_{2}$, as explained in Reference 17.

We assume that the transition probabilities in both the model of incidence and the model of mortality only depend on a subject's index through corresponding covariates. Subjects are assumed to be independent such that the posterior distribution of future events given $\theta_{p},\theta_{1},\theta_{2}$ factorizes into joint posteriors of *S*_1, *i*_, *T*_1, *i*_ for *i* ∈ *I*_fut_ and of *S*_2, *i*_, *T*_2, *i*_ for *i* ∈ *M*_fut_.

We use the algorithm below with two loops that produces a total of *m* × *n* samples from the posterior distribution. We chose to locate the imputation of missing data and estimation of each model component in the outer loop as they typically are the most computationally intensive parts. The inner loop is justified only when the corresponding within‐loop computations are significantly faster than those in the outer loop, and can in that case be used to relatively quickly generate more simulations. Indices *a* and *b* of the loops are omitted for notational convenience.

Algorithm 1 **Estimation and simulation algorithm**

1

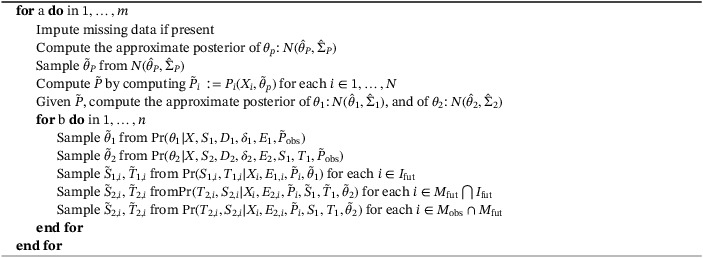

## 3 APPLICATION TO PROSTATE CANCER TESTING

Prostate cancer is often grouped into five ordered risk categories (low, intermediate, and high risk and regionally metastatic or distant metastatic) with increasing risk of progression and death.^18^, ^19^ In short, these categories are defined by serum levels of PSA, histopathological assessment of needle core biopsies by use of the Gleason classification, and TNM staging, which is based on the tumour size (T), involvement of lymphatic nodes (N), and presence of distant metastases (M). The intensity of diagnostic activity can be determined by how likely men without diagnosis are tested using the PSA test, biopsies, and/or procedures for T staging. Therefore, the diagnostic activity is interpreted in a broader sense and not only as the frequency of PSA testing.

Because PSA tests can detect prostate cancer in asymptomatic men, the incidence of low‐risk prostate cancer increases and the incidence of metastatic prostate cancer decreases in populations undergoing frequent PSA‐testing.^1^, ^2^, ^3^, ^4^, ^5^ However, the cost‐benefit balance of PSA testing for detection of prostate cancer remains debated.^6^ Two large randomized clinical trials have shown statistically significant decreases in prostate cancer specific mortality after 11 to 14 years of follow‐up in screened vs unscreened men, with a 21% relative decrease in the European Randomized Study of Screening for Prostate Cancer (ERSPC) trial as a whole^20^ and a 44% relative decrease in the Swedish part of ERSPC.^21^ However, many clinical scientists argue that additional biomarkers for early detection of prostate cancer are needed before population‐based prostate cancer screening should be introduced.^22^, ^23^

The uptake of PSA testing in Sweden has been substantial despite the absence of national recommendations, and large variations between counties in terms of uptake and incidence have been observed.^24^, ^25^ The diagnostic activity in Stockholm region in terms of PSA testing and biopsies has been described in detail.^26^, ^27^

### 3.1 Key assumptions

We defined the risk categories low and intermediate as *lower risk*, and high risk, regionally metastatic and distant metastases as *higher risk*. Time was discretized in units of 1 year. The experimental units were defined by region of residence and birth year. We assumed that it was sufficient to include the history of the proxy for diagnostic activity up to 20 years back as mean lead time in prostate cancer screening has been estimated to be of the order of 10 years.^12^, ^28^ In addition, we assumed that treatment options and strategies and the effect of treatment on survival from diagnosis have been constant since 2016. We specified two scenarios: (A) continued high diagnostic activity as in Stockholm region during 2010 and (B) low diagnostic activity as in Stockholm 1996.

### 3.2 Materials

Data on 236 506 men with prostate cancer diagnosed between 1970 and 2016 were primarily obtained from Prostate Cancer data Base Sweden (PCBaSe).^29^, ^30^ Men diagnosed between 1970 and 1991 were only included if alive at 1 January 1992. Data on 842 536 prostate cancer‐free men (hereon denoted by Controls) matched by birth year and region of residency to each prostate cancer case were obtained from the same source. Data linkages through the use of the Swedish personal identity number in PCBaSe have been performed between the National Prostate Cancer Registry of Sweden (NPCR) and other health care registries and demographic databases as previously described.^31^ NPCR is a nationwide clinical cancer register that captures data on cancer characteristics according to the TNM classification, Gleason grading,^32^, ^33^ serum level of PSA, diagnostic work‐up and primary treatment. Other health care registers include the Patient Register, the Prescribed Drug Registry, the Swedish Cancer Registry, the Cause of Death Registry, and the Longitudinal Integration Database for Health Insurance and Labour Market Studies (LISA). Information on other cancer diagnoses was retrieved from the Cancer Registry, date and cause of death were retrieved from the Cause of Death Registry, and educational level and marital status were retrieved from the LISA database. Data on diagnostic activity in terms of PSA testing, biopsies, and assessment of TNM stages were not available among all Swedish men, neither on an individual nor on a regional level.

Comorbidity at date of diagnosis was classified into four categories according to the Charlson Comorbidity Index (CCI), which is a weighted sum of a number of diagnostic codes excluding prostate cancer in the Patient Registry as previously described.^34^

Since 1998, NPCR captures 98% of all of incident prostate cancer cases in Sweden compared to the Swedish Cancer Registry to which registration is mandated by law.^35^ Therefore, data on men registered in the Swedish Cancer Registry but not present in the NPCR were also obtained.

We used a modified version of the National Comprehensive Cancer Network 5‐tiered risk categorization, previously described in Reference 19. Recent findings^36^, ^37^ have indicated that a PSA cut‐off of 100 ng/mL is suboptimal for indicating metastatic disease, and therefore we defined distant metastases as either M stage M1 or PSA ≥ 500 ng/mL. Consequently, regionally metastatic is defined as either T stage T4 or N stage N1 or PSA between 50 and 500 ng/mL.

Demographic data on the total male population in Sweden during the period and a prediction of future demography of Sweden were obtained from Statistics Sweden.^38^, ^39^ The prognosis was used to determine the number of men alive at the age of 40 each year on January 1. These data are not available by region, so it was assumed that the proportion of men is constant across counties for future years. The final dataset contained both completely and partially observed variables such as birth date, region, start and end of follow‐up, status at end of follow‐up, risk category at diagnosis, time of diagnosis, and death. The experimental units were defined by a combination of birth year (1892‐2020) and the 21 counties in Sweden (Norrbotten, Jämtland, Västerbotten, Västernorrland, Dalarna, Gävleborg, Uppsala, Värmland, Örebro, Västmanland, Södermanland, Stockholm, Gotland, Östergötland, Jönköping, Kalmar, Västra Götaland, Halland, Blekinge, Kronoberg and Skåne).

### 3.3 Imputation of missing data

Missing data occurred only on men with prostate cancer diagnosis and the primary variable of interest with missing data was the risk category. Therefore, the imputation models only imputed and depended on data on men with prostate cancer. The following variables had complete data: age at diagnosis, year of diagnosis, time of follow‐up, and cause of death or censoring. Almost all men had recorded region of residence at time of diagnosis. We imputed the missing data through a sequence of marginal models using chained equations implemented in R by the MICE algorithm.^40^ Variables apart from region of residence, dates (eg, date of diagnosis), cause of death, and those appearing in the definition of the risk categorization, were only used to improve the accuracy of the imputation. The amount of missing data and reason for missingness of the data varied across calendar year and region partly due to historical reasons.^19^ Data was considered missing at random (MAR) apart from M stage which was imputed using logistic regression but with a modified algorithm to model a nonignorable missing data mechanism.^41^ A similar imputation model has previously been used and described in.^37^, ^42^

### 3.4 Model specification and estimation

For each competing risk in each model component, the parameters were estimated using separate logistic regression models where subjects experiencing competing events were artificially censored,^16^ using REML. Parameter estimation was performed using R‐package *mgcv*.

The model of the proxy for diagnostic activity included age, calendar year and region of residence as covariates. Specifically, the linear predictor was assumed to be in the form $\sum_{i=1}^{21}f_{i}(a,y),$ where *i* = 1, … , 21 representing the 21 counties in Sweden. Counties are assumed to be independent, so estimation was stratified by region. Possible effects of the Stockholm 0 trial and Göteborg screening trial^43^ in certain ages and years were modelled using an indicator of the respective age and year combinations, and the multidimensional splines were specified separately for the two levels of the indicator in each of the two counties. The proxy before to 1985 was assumed to be the smallest of the proxy in counties that had low incidence during 1992 (Blekinge, Gävleborg, Skåne, Stockholm, Södermanland and Värmland) since the age standardized incidence in men aged 50 to 74 was even lower before 1992.^24^ We used age‐dependent linear interpolation between 1985 and 1992. After 2016, an assumption on the proxy was needed. We specified two scenarios: one with continues high diagnostic activity as in Stockholm region during 2010 and one with limited diagnostic activity as in Stockholm 1996. These targets were reached in 2020 in all counties with linear interpolation from 2016 and 2020. The history of the diagnostic activity was assumed to be fixed from age 40.

The five separate logistic regression models governing the probability of being diagnosed with risk category 1 to 5 vs staying alive had linear predictors on the form $f_{c}(a,\log(\lambda (X_{i},\theta_{p},a)))+f_{h}(a,\log(\lambda (X_{i},\theta_{p},a-1)),\ldots ,\log(\lambda (X_{i},\theta_{p},a-20)))$, where *f*_*c*_ was a smooth function of age *a* and the proxy at current time $\lambda_{a}$ while *f*_*h*_ were a smooth function of current age and the proxy at past times. The model governing the risk of death vs staying alive had a linear predictor on the form *f*(*a*) + *g*(*y*), for smooth functions *f* and *g* of age (*a*) and calendar year (*y*) correspondingly. It was only possible to distinguish censoring and death for men without a diagnosis among controls, as detailed data on time of censoring were not available for the whole population. Consequently the estimation of parameters was performed on this subset of the data. Subjects in this subset of the data had a randomized start of follow‐up and did not contribute to the likelihood before that point.^44^

The two separate logistic regression models governing the probability of death by prostate cancer or other causes vs staying alive for men with a diagnosis had linear predictors of the form $\sum_{s=1}^{5}f_{s}^{1}(a,t)+f_{s}^{2}(a,y)+f_{s}^{3}(a_{c})+f_{s}^{4}(y_{c})$, where $f_{s}^{1}(a,t)$ is a two‐dimensional spline representation of a function of age at diagnosis *a* and time since diagnosis *t*, for risk categories *s* = 1, … , 5, and the other terms were similar two‐, respectively, one‐dimensional functions of year of diagnosis *y*, current age *a*_*c*_ and current year *y*_*c*_. Note that year and cause of death was assumed to be conditionally independent of the proxy of diagnostic activity given year of diagnosis and risk category.

### 3.5 Simulation

First, missing data for men with prostate cancer were imputed using *m* = 20 imputations, see Algorithm 1. Simulations were performed *n* = 5 times for each multiple imputed data sets, rendering a total of 100 simulations. For each model component, new coefficients were drawn from a multivariate normal distribution with the point estimates as mean and the estimated Bayesian posterior covariance matrix provided by the *vcov.gam* method as covariance matrix.

### 3.6 Validation

Incidence and mortality models were estimated using data until 31 December 2012, while the model of the proxy for diagnostic activity is estimated using all data. Two simulations—one starting in 1992 and one starting in 2013 with both ending in 2016—were performed to validate the model given the imputed data and the proxy for diagnostic activity. In addition, the model performance is also evaluated after being estimated on the full dataset. Note that these simulations were performed only conditional on the number of men at risk of being diagnosed in each experimental unit in the beginning of the simulation and on the number of men becoming 40 years old and therefore at risk sometime during the simulation.

### 3.7 Analysis of results

The analysis of observed and simulated data consists of estimating the number of men diagnosed and the proportion of men diagnosed per men alive without diagnosis, number of deaths by prostate cancer and other causes, the corresponding proportions per number of men alive with diagnosis, the incidence rate of prostate cancer death (IR), which is the cumulative sum of prostate cancer deaths divided by the total number of person years for all men from the start of the simulation (2017), and the incidence rate ratio (IRR), which is the ratio of the incidence rates extracted from each of the two scenarios. Estimates were pooled using a set of modified parameter pooling rules^45^ (Rubin's rules) adapted for nested simulations.^46^ The statistical analysis was performed using R 3.4.1 RC.^47^ The Research Ethics board in Uppsala, Sweden approved the use of PCBaSe 4.

## 4 RESULTS

### 4.1 Understanding the model

**FIGURE 2:**
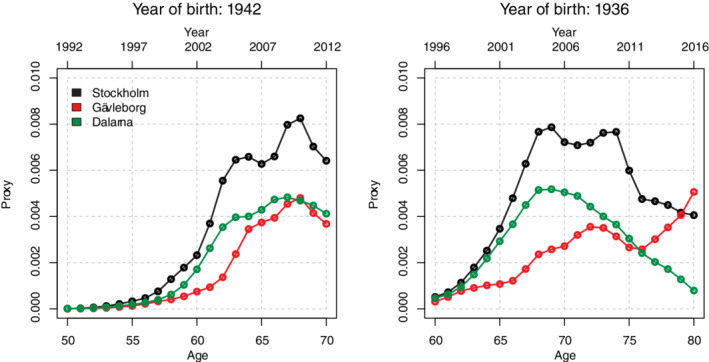
Example of the proxy for diagnostic activity in terms of the probability of being diagnosed with lower risk disease vs staying alive without diagnosis [Colour figure can be viewed at wileyonlinelibrary.com]

We illustrate the proxy for diagnostic activity for two birth cohorts and three counties in Figure 2. For each model of incidence, the proxy was used given a specific calendar year: the proxy at current year combined with current age, and the history of the proxy 1 to 20 years back combined with the age at those times. In Figure S1 the proxy is illustrated for all birth cohorts and 10 counties. Uptake of testing varied largely across ages, counties, and calendar periods. Some counties (eg, Gävleborg) had a late uptake, whereas others (eg, Kalmar) had an early uptake. A few (eg, Dalarna and Örebro) had a peak around 2010, after which diagnostic activity decreased. An example of the effect of different diagnostic activity patterns (scenario A vs B) on the hazard and the cumulative incidence for men in Stockholm followed between 2017 and 2060 from age 47 to 90, is shown in Figure S2A,B.

### 4.2 Model validation

**FIGURE 3:**
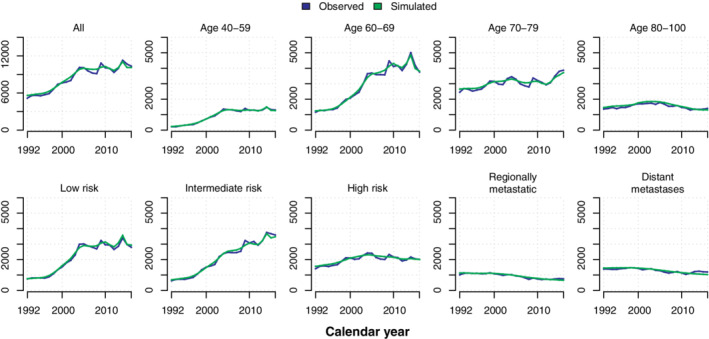
Observed and simulated number of prostate cancer diagnoses by risk category and age [Colour figure can be viewed at wileyonlinelibrary.com]

**FIGURE 4:**
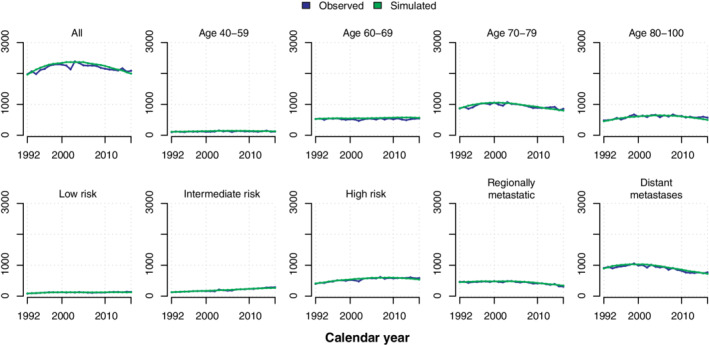
Observed and simulated number of prostate cancer deaths by risk category and age [Colour figure can be viewed at wileyonlinelibrary.com]

Observed and simulated number of diagnoses and prostate cancer deaths are shown in Figures 3 and 4. Incidence and mortality models were estimated on data until 31 December 2012. The simulated incidence and prostate cancer mortality (green) curve followed the observed incidence (blue) across calendar time in each risk category. Very similar results were observed when the simulation was started in 2013 (data not shown). A fully stratified analysis including the risk of death by other causes, can be found in Figures S3 to S5. Similar trends were observed for other cause mortality.

**FIGURE 5:**
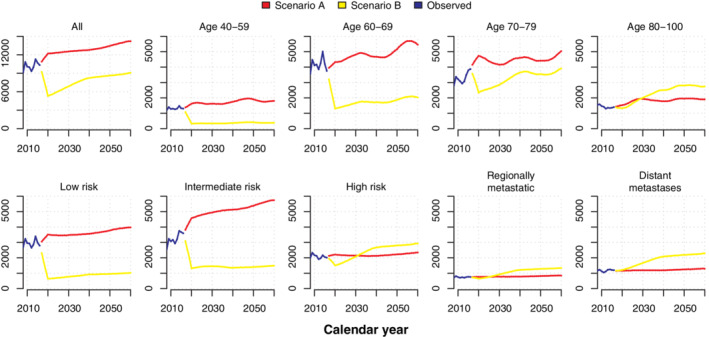
Simulated number of prostate cancer diagnoses between 2017 and 2060 by risk category and age under scenarios (A) continued high diagnostic activity as in Stockholm during 2010 and (B) low diagnostic activity as in Stockholm 1996. [Colour figure can be viewed at wileyonlinelibrary.com]

**FIGURE 6:**
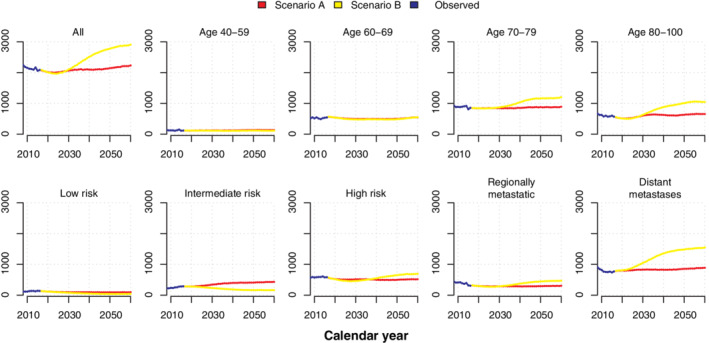
Simulated number of prostate cancer deaths between 2017 and 2060 by risk category and age under scenarios (A) continued high diagnostic activity as in Stockholm during 2010 and (B) low diagnostic activity as in Stockholm 1996 [Colour figure can be viewed at wileyonlinelibrary.com]

### 4.3 Prediction until 2060

**FIGURE 7:**
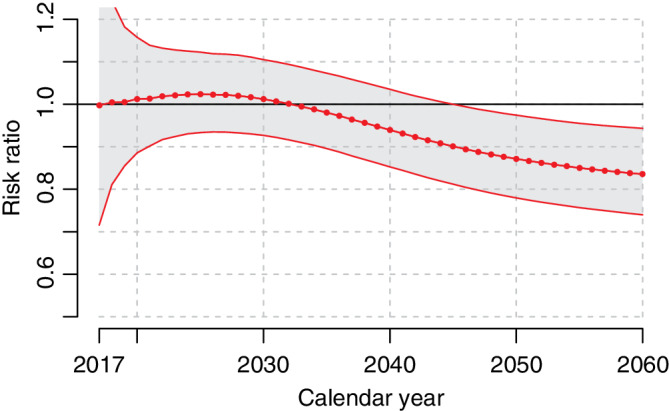
Incidence rate ratio of prostate cancer death between scenarios (A) continued high diagnostic activity as in Stockholm during 2010 and (B) low diagnostic activity as in Stockholm 1996. Dotted line indicate yearly point estimate, and solid lines indicate corresponding approximate 95% confidence interval [Colour figure can be viewed at wileyonlinelibrary.com]

A prediction of incidence and mortality between 2017 and 2060 under scenarios A and B is shown in Figures 5 and 6, and a fully stratified analysis can be found in Figures S6 and S7. All models were estimated on the full dataset. Scenario A (frequent testing) revealed a higher incidence of low and intermediate risk prostate cancer starting in 2017; after 2030, the incidence of metastatic prostate cancer was lower. Compared to scenario B, scenario A had fewer prostate cancer deaths (Figure 6) and ultimately a reduction in yearly IRR of prostate cancer death among all men (Figure 7): for example, after after 11 years (2027) the IRR was 1.02 (95% CI: 0.93‐1.12) and after 44 years (2060) the IRR was 0.84 (95% CI: 0.74‐0.94).

## 5 DISCUSSION

### 5.1 Main results

We developed a model of the effect of diagnostic activity on disease incidence and mortality adapted for real‐life population‐based data. The model (named PRISM‐PC) was applied to data from PCBaSe and demographic data. Incidence of low and intermediate risk prostate cancer was used as a proxy for diagnostic activity. PRISM‐PC reconstructed the observed number of diagnoses and deaths in all strata defined by age groups, calendar years, and risk categories. Next, predictions were produced under two scenarios—high and low diagnostic activity—and these differed in terms of the number of diagnoses and distribution of risk category at diagnosis. For example, the number of men diagnosed with metastatic disease and the number of prostate cancer deaths were lower in the scenario of high diagnostic activity.

### 5.2 Comparison with other simulation models

Several studies have attempted to use models to produce prognoses of effects of cancer testing and screening by simulating clinical trials (see below). However, unlike these models, PRISM‐PC compares current clinical practice against historical practice in a given country or predicts incidence and mortality in a real‐life situation rather than emulating clinical trials, investigating appropriate testing strategies, or modelling the natural history of prostate cancer. In addition, PRISM‐PC depends on all diagnostic activities, and not just the frequency of PSA testing.

Examples of other simulation models include the MISCAN‐COLON simulation model for the evaluation of colorectal cancer screening,^10^ a stochastic simulation model of ovarian cancer screening,^13^ the Wisconsin breast cancer epidemiology simulation model^14^ implemented to explore cost effectiveness of digital mammography breast cancer screening,^15^ and models of effects of PSA screening on prostate cancer mortality.^48^ More recently, the FHCRC model of PSA screening^7^ was applied using Swedish registry data on prostate cancer.^8^ Several instances of the MISCAN‐model^9^ have used various data sources.^10^, ^11^, ^12^

The above models require explicit assumptions about the epidemiology and natural history of the corresponding cancer. As an example, the FHCRC model^8^ describes the natural history before diagnosis using PSA trajectories, and PSA testing and biopsy compliance. Each submodel had parameters that require calibration, which is important for the validity of subsequent analyses.^49^ For example, parameters that require calibration govern the natural history, the screening effect of incidence sub‐model, and the survival from diagnosis. The submodel of survival from diagnosis was calibrated against 10‐ and 15‐year survival estimated on data from PCBaSe. Death by other causes among men with and without prostate cancer was based on data from standard male life tables. Furthermore, the models used explicit assumptions on the lead time effect of earlier detection by screening survival from diagnosis.

In comparison, PRISM‐PC requires no explicit assumptions about compliance, properties of tests, or the natural history of the disease, that is, our model is designed to approximate real‐life scenarios assuming as little as possible on the complex dynamics between age, compliance, sensitivities of tests, age and state of latent disease. PRISM‐PC is still flexible enough to accommodate changes in incidence, mortality, and diagnostic activity. The model of risk of death by other causes in men with prostate cancer was estimated using data on men with prostate cancer without assuming that this risk is the same for men in general. We used look‐back of up to 20 years, which is far longer than the previously estimated lead time effect of screening. However, we did not explicitly model lead time effects on survival from diagnosis given risk category primarily because it is difficult separating these from the effects of introduction of new and/or improved treatments.^42^ Therefore, the primary lead time effect on survival in PRISM‐PC is determined by the potentially earlier time of diagnosis and lower risk category at diagnosis, and we assumed that the effect of treatment on survival from diagnosis was constant from 2016.

### 5.3 Previous studies on prostate cancer testing and screening

Although PRISM‐PC was not designed to emulate clinical trials, it is still possible to compare outcomes between scenarios in terms of IRRs for example. Two large population‐based randomized clinical trials have shown statistically significant decreases in prostate cancer specific mortality after 11 and up to 14 years of follow‐up in screened vs nonscreened men: the ERSPC trial revealed a 21% decrease as a whole^20^ and a 44% decrease in the Swedish part of ERSPC.^21^ However, we saw a slightly larger IRR that decreased slowly and ultimately reaching a 26% decrease, IRR 0.84 (95% CI: 0.74‐0.94), after 44 years of follow‐up.

The biggest difference between our simulation and the ERSPC trial is that the ERSPC trial was a clinical trial and our simulations approximate an ecological study of frequent vs infrequent unorganized opportunistic prostate cancer testing and not formalized population‐based screening. We included all men between 40 and 100 years old instead of 55 to 69 as in the trials. In the ERSPC trial men were invited to be tested every 4 years (2 years in Sweden) while, for example, the 1‐year prevalence proportion of PSA‐testing in Stockholm in 2011 was 17%, 27%, and 31% for men aged 50 to 59, 60 to 69, and 70 to 79, respectively.^26^ These differences are likely to explain the difference in prostate cancer mortality given that radical treatment in newly detected prostate cancer was part of the design in the trial.

### 5.4 Strengths and limitations

Strengths of PRISM‐PC include that few assumptions are made on the disease dynamics in terms of the relationship between age, natural history, history of testing, test properties, and compliance. The hazards were specified using flexible functions whose shape to large extent could be determined by data, instead of assuming Weibull or exponential distributions. We used detailed data from high‐quality nation‐wide population‐based registers covering a wide age range (40‐100 years men). For example, prostate cancer was extremely rare before the age of 40 (25 men in our dataset), and it was uncommon that men above the age of 100 were diagnosed (6 men in our dataset). Missing data was handled using modern imputation methods, and, due to the nature of posterior sampling, uncertainty about parameters and outcomes generated from both the missing data imputation and each step in the simulation could be accounted for.

The use of a time discretization window of one year is reasonable because the aim is to model the effect of changes in diagnostic activity over longer periods of time corresponding to changes in general health care strategies, and not to model any seasonal variation of incidence over a year, for example, due to vacations or seasonal variations in the health care structure.^50^ Possibly, the risk of death among diagnosed men could more accurately be modeled using shorter a time window, and one could for example use time units of 1 month or 1 week for this submodel.

It is natural to define a proxy of the diagnostic activity using the incidence of *lower risk* (ie, low and intermediate risk) prostate cancer because such disease is more often asymptomatic^42^, ^51^ than higher risk disease, and because the incidence has increased threefold between 1992 and 2016, Figure S3, in line with the increase in use of PSA testing.^25^ However, there are several others risk categorization systems, and some could be more predictive of prostate cancer death. A future version of PRISM‐PC could implement another risk categorization system.

A limitation of our study is the use of a proxy for the measure of diagnostic activity either directly on individual or group level. When using a proxy, as we have done, it is important that it is related to diagnostic activity and that the underlying risk of disease is constant over calendar time and experimental units and only varies with age. Changes in exposure to unknown etiological factors could affect the underlying risk. If not, there has to be enough data available such that this can be adjusted for when constructing the proxy or it will be confounded with effects of changes in diagnostic activity. The proxy is also difficult to interpret directly in terms of diagnostic activity, which is important when trying to understand the specifications of our scenarios in the simulation. We plan to extend the model by use of data on PSA testing and biopsies to remedy this, as these types of data may become more available in the future.

Another limitation of our study is that the prediction of year and cause of death relies on the assumption that treatment options and strategies remain the same during the period of interest, which is unlikely when the study period is very long. The definition of the risk categories is assumed to be fixed; however, changes in Gleason grading have occurred that result in an inflation, which is a limitation in temporal comparisons.^42^, ^52^ Diagnostic activity have changed over time (eg, the introduction of new imaging techniques) with and ensuing change in risk categories. The effect of risk category on survival could also be affected by within‐stage migration due to earlier detection, which our model does not adjust for. However, these issues are inherent for all observational studies.

Another limitation of PRISM‐PC is the question if the demographic prognosis are reliable. We used a prognosis from Statistics Sweden, that is the national authority responsible for official statistics.

Moreover, as demographic data are only available on an aggregate level, it was not possible to track individuals. When a man moving from one region to another we implicitly had to assume that he would obtain the history of the proxy in the latter region at the year of relocation, and this could possibly cause a diluting effect on model estimates, making the association between diagnostic activity and incidence weaker. This could happen when, for example, previously tested and nondiagnosed men move to region where the diagnostic activity was lower. During 2019 in Sweden, around 226 000 persons moved across region boarders, and two‐thirds of these moves were performed by persons 18 to 39 years old.^53^ We caution that the effect of in and out moves between different regions could be more influential when the prevalence of latent disease detectable by a screening test is high in the subpopulation where migration is common and where the diagnostic activity varies between regions and subpopulations. If incidence of lower risk disease is homogeneous across regions but the incidence of more advanced disease is not, then this could be a sign of effects of migration, or alternatively, if the model components for incidence of more advanced disease (eg, metastatic) fits the data equally well when excluding the proxy as when including it. In our study, we saw that the incidence of lower risk disease varies between regions, age groups and calendar periods, and so does the incidence of more advanced disease, and when excluding the proxy, each of the incidence model components performed significantly worse with a clear dependency between residuals and calendar time. Thus, only a rather small fraction of men in the age range of PRISM‐PC moved to another region and that this had little effect on the model.

We further argue that ignoring moves between regions is less of an issue when producing prognoses, especially if the targeted future diagnostic activity is identical across geographical regions (eg, counties). In this case, migration between counties only affects the prediction during a transition period. Immigration could also have a diluting effect of unknown direction, depending on the immigrant's history of diagnostic activity. Between 2002 and 2018, 95% of all immigrating men who moved to Sweden were younger than 55.^54^

### 5.5 Possible applications and future work

We plan to use PRISM‐PC for multiple purposes. The first purpose is to evaluate the effects of the observed increased diagnostic activity between 1992 and 2016 compared with a hypothetical more restrictive test policy. The second purpose is to produce predictions based on various levels of diagnostic activity, and to study heath economics and quality of life. Other possible model extensions include the modeling of detailed disease trajectories, and future work includes combining PRISM‐PC with an already existing state transition model given prostate cancer diagnosis.^55^ This extension will allow for prediction of prevalence under various assumptions on changes in both diagnostic activity and treatment strategies.

### 5.6 Conclusion

We constructed a simulation model of disease incidence driven by diagnostic activity that requires no explicit assumptions on the natural history of the disease and properties of clinical tests, but requires multiple risk categories and a proxy for diagnostic activity. The model was estimated using Swedish nation‐wide population‐based data on prostate cancer and no parameter calibration was required. It was flexible enough to accurately reconstruct observed incidence and mortality. We used the model to compare effects of high vs limited diagnostic activity under the assumption that effects of treatments on survival were constant with respect to future incidence and prostate cancer mortality where limited diagnostic activity leads to higher incidence of more advanced disease and higher prostate cancer mortality.

Thus, it is possible to approximate the dynamics of age, incidence, mortality, and diagnostic activity in a real‐life setting with accuracy without modelling the latent disease trajectory, properties of tests, and the effect of testing on lead time and survival.

## CONFLICT OF INTEREST

The authors declare no potential conflict of interests.

## Supporting information

Figure S1. Example of the proxy for diagnostic activity in terms of the probability of being diagnosed with lower risk disease vs staying alive without diagnosis. Yellow indicates smaller hazard and red larger hazard. Vertical grey line indicates the start of the simulation (2017). Scenario A (continued high diagnostic activity as in Stockholm county during 2010) is exemplified in terms of the proxy to the right of this line

Figure S2. (A) The effect of continued high diagnostic activity as in Stockholm during 2010 (scenario A) vs low diagnostic activity as in Stockholm 1996 (B) on the probability of being diagnosed by risk stage at diagnosis

Figure S3. Observed and simulated incidence by risk category and age groups. Incidence and mortality models estimated on data until 31 December 2012

Figure S4. Observed and simulated prostate cancer mortality by risk category and age groups. Incidence and mortality models estimated on data until 31 December 2012

Figure S5. Observed and simulated other cause mortality among men with prostate cancer by risk category and age groups. Incidence and mortality models estimated on data until 31 December 2012

Figure S6. Simulated incidence between 2017 and 2060 by risk category and age under scenarios (A) continued high diagnostic activity as in Stockholm during 2010 and (B) low diagnostic activity as in Stockholm 1996. Models estimated on all data

Figure S7. Simulated prostate cancer mortality between 2017 and 2060 by risk category and age under scenarios (A) continued high diagnostic activity as in Stockholm county during 2010 and (B) low diagnostic activity as in Stockholm 1996. Models estimated on all data

## Data Availability

These data are not publicly available due to concerns of confidentiality. However, external researchers may apply for limited remote access to data. Users will be charged for software licences, administration, and data management. For more information, contact npcr@npcr.se.
