# Supplementary material for: Simulation model of disease incidence driven by diagnostic activity
Source: Stat Med. 2020 Nov 25;40(5):1172–88. doi: 10.1002/sim.8833 (PMC7894333; doi:10.1002/sim.8833)
Supplement: Supplementary file 1 — Figure S1. Example of the proxy for diagnostic activity in terms of the probability of being diagnosed with lower risk disease vs staying alive without diagnosis. Yellow indicates smaller hazard and red larger hazard. Vertical grey line indicates the start of the simulation (2017). Scenario A (continued high diagnostic activity as in Stockholm county during 2010) is exemplified in terms of the proxy to the right of this line [file SIM-40-1172-s001.pdf]

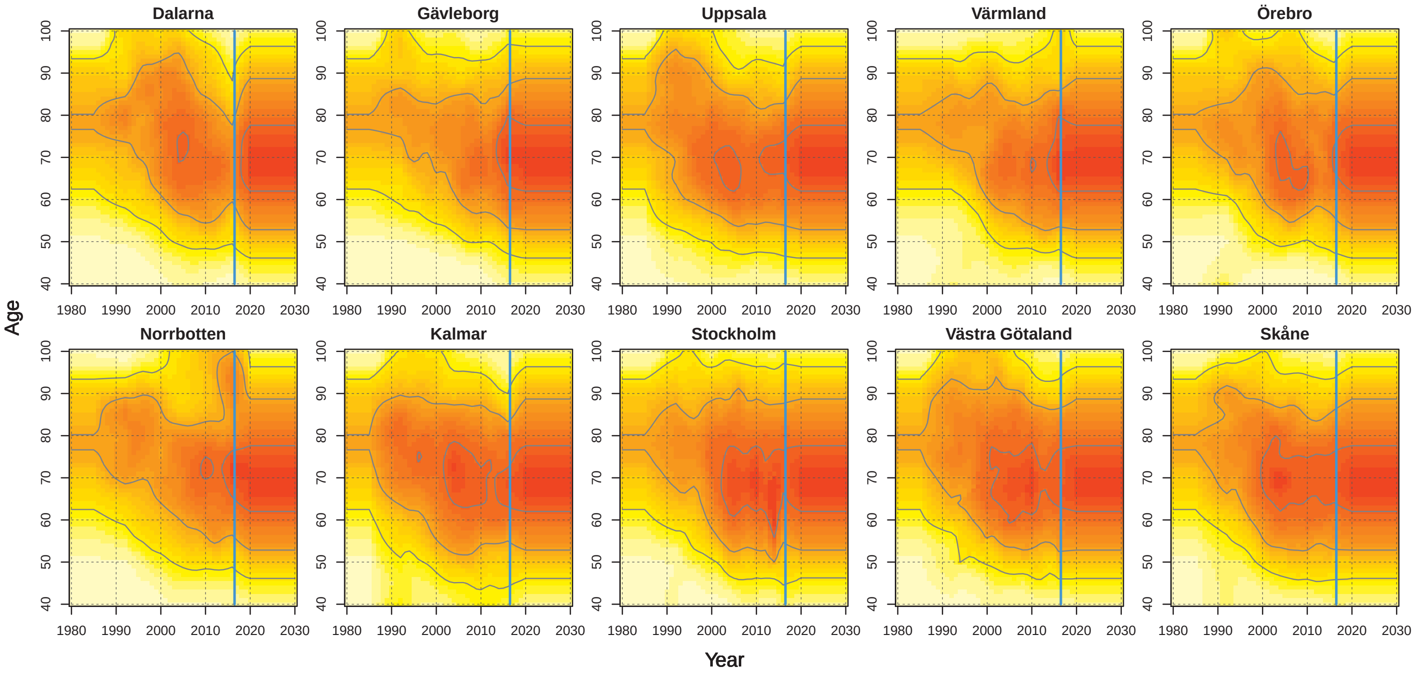

Supplementary Figure 1. Example of the proxy for diagnostic activity in terms of the probability of being diagnosed with lower risk disease vs staying alive without diagnosis. Yellow indicates smaller hazard and red larger hazard. Vertical grey line indicates the start of the simulation (2017). Scenario A (continued high diagnostic activity as in Stockholm county during 2010) is exemplified in terms of the proxy to the right of this line.
