# Supplementary material for: Simulation model of disease incidence driven by diagnostic activity
Source: Stat Med. 2020 Nov 25;40(5):1172–88. doi: 10.1002/sim.8833 (PMC7894333; doi:10.1002/sim.8833)
Supplement: Supplementary file 2 — Figure S2. (A) The effect of continued high diagnostic activity as in Stockholm during 2010 (scenario A) vs low diagnostic activity as in Stockholm 1996 (B) on the probability of being diagnosed by risk stage at diagnosis [file SIM-40-1172-s002.pdf]

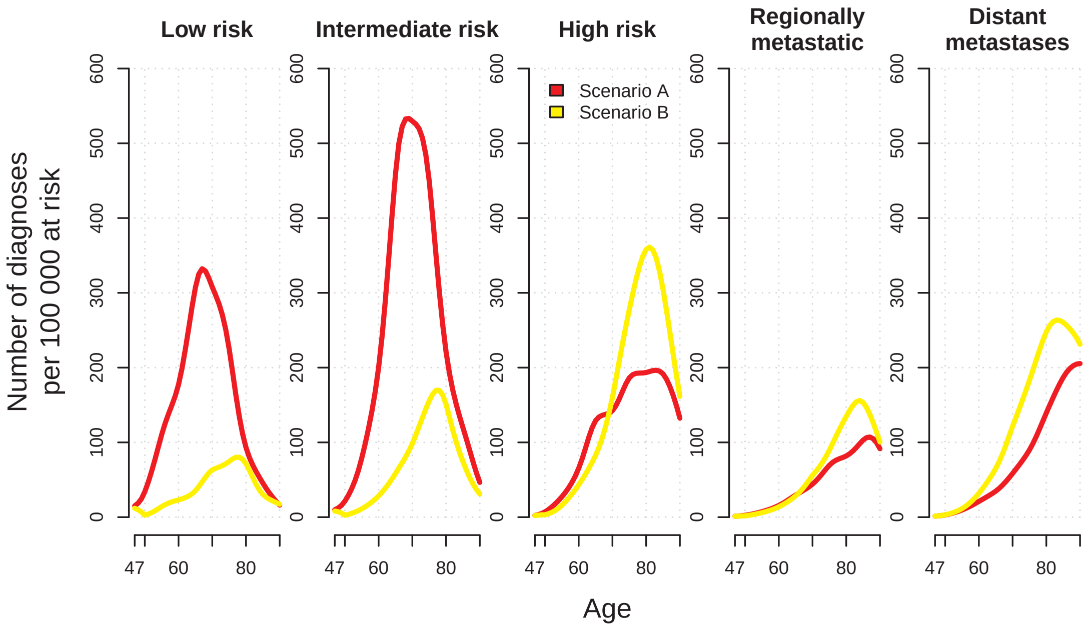

Supplementary Figure 2a. The effect of continued high diagnostic activity as in Stockholm during 2010 (scenario A) vs low diagnostic activity as in Stockholm 1996 (B) on the probability of being diagnosed by risk stage at diagnosis. Supplementary

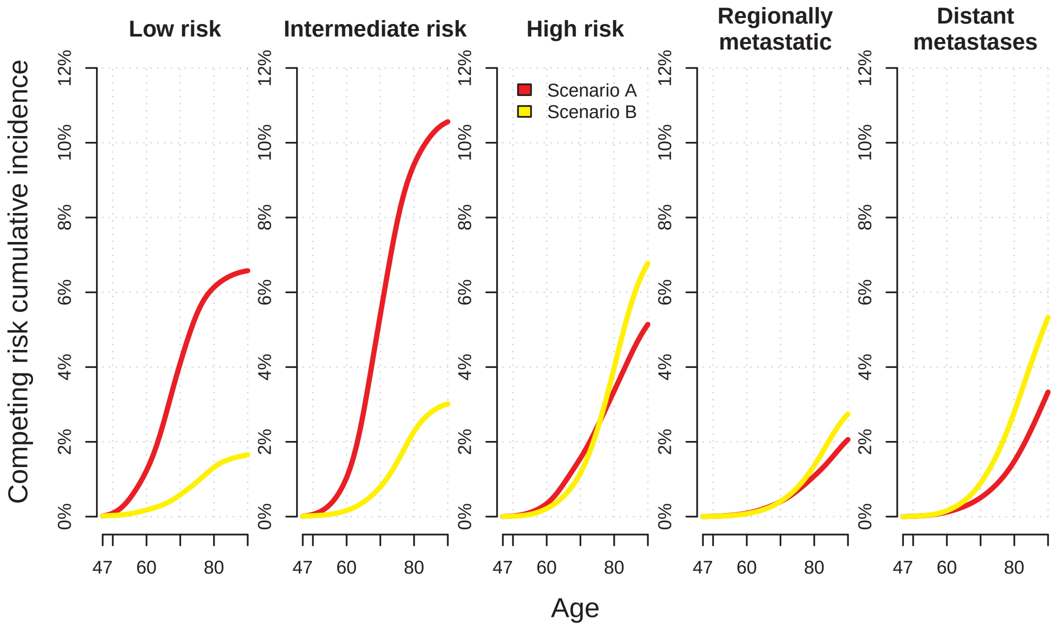

Figure 2b. The effect of continued high diagnostic activity as in Stockholm during 2010 (scenario A) vs low diagnostic activity as in Stockholm 1996 (B) on the cumulative incidence of being diagnosed by risk stage at diagnosis.
