# Supplementary material for: Simulation model of disease incidence driven by diagnostic activity
Source: Stat Med. 2020 Nov 25;40(5):1172–88. doi: 10.1002/sim.8833 (PMC7894333; doi:10.1002/sim.8833)
Supplement: Supplementary file 4 — Figure S4. Observed and simulated prostate cancer mortality by risk category and age groups. Incidence and mortality models estimated on data until 31 December 2012 [file SIM-40-1172-s004.pdf]

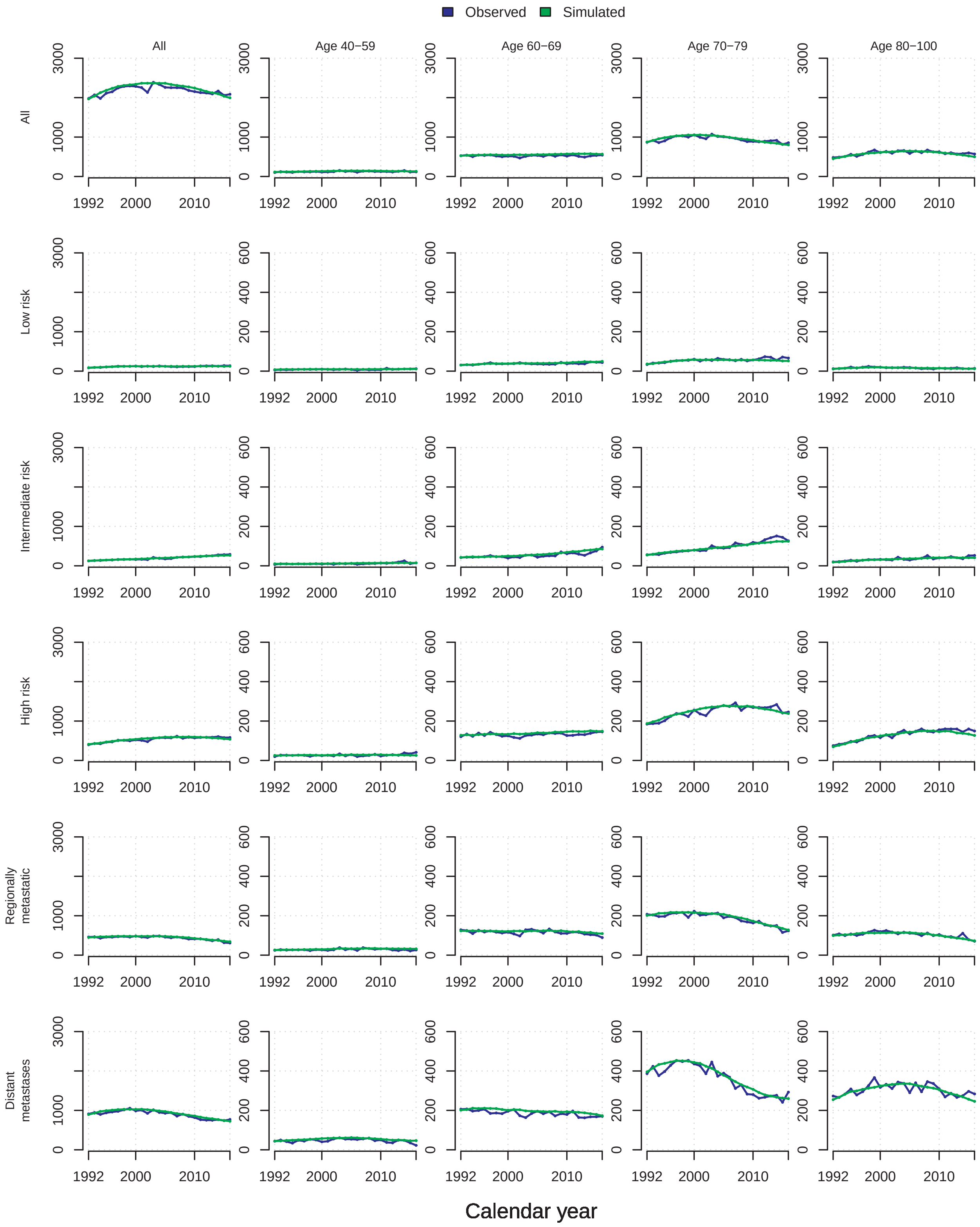

Supplementary Figure 4. Observed and simulated prostate cancer mortality by risk category and age groups. Incidence and mortality models estimated on data until 31 December 2012.
