# Supplementary material for: Simulation model of disease incidence driven by diagnostic activity
Source: Stat Med. 2020 Nov 25;40(5):1172–88. doi: 10.1002/sim.8833 (PMC7894333; doi:10.1002/sim.8833)
Supplement: Supplementary file 5 — Figure S5. Observed and simulated other cause mortality among men with prostate cancer by risk category and age groups. Incidence and mortality models estimated on data until 31 December 2012 [file SIM-40-1172-s005.pdf]

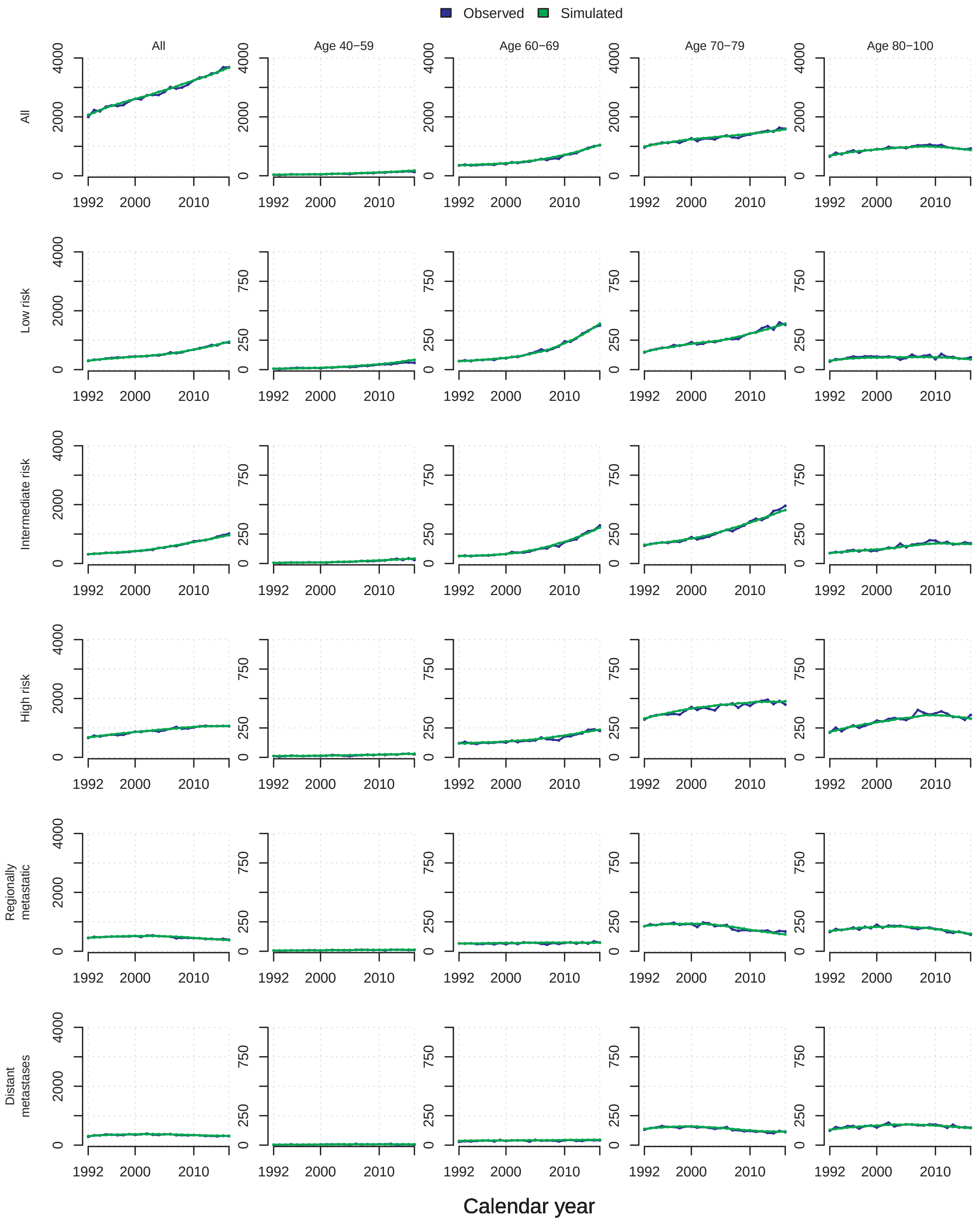

Supplementary Figure 5. Observed and simulated other cause mortality among men with prostate cancer by risk category and age groups. Incidence and mortality models estimated on data until 31 December 2012.
