# Supplementary material for: Simulation model of disease incidence driven by diagnostic activity
Source: Stat Med. 2020 Nov 25;40(5):1172–88. doi: 10.1002/sim.8833 (PMC7894333; doi:10.1002/sim.8833)
Supplement: Supplementary file 6 — Figure S6. Simulated incidence between 2017 and 2060 by risk category and age under scenarios (A) continued high diagnostic activity as in Stockholm during 2010 and (B) low diagnostic activity as in Stockholm 1996. Models estimated on all data [file SIM-40-1172-s006.pdf]

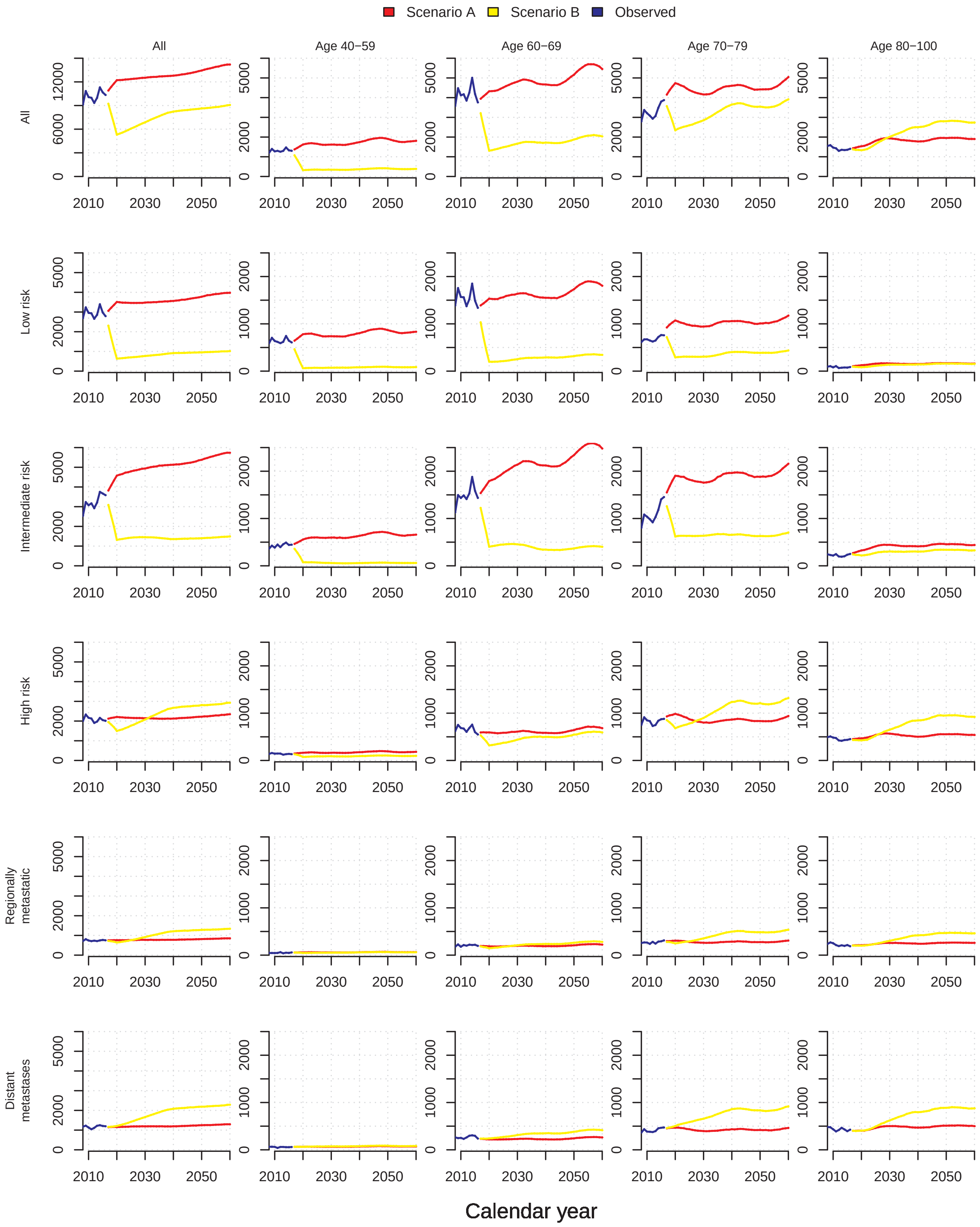

Supplementary Figure 6. Simulated incidence between 2017 and 2060 by risk category and age under scenarios (A) continued high diagnostic activity as in Stockholm during 2010 and (B) low diagnostic activity as in Stockholm 1996. Models estimated on all data.
