# Supplementary material for: Simulation model of disease incidence driven by diagnostic activity
Source: Stat Med. 2020 Nov 25;40(5):1172–88. doi: 10.1002/sim.8833 (PMC7894333; doi:10.1002/sim.8833)
Supplement: Supplementary file 7 — Figure S7. Simulated prostate cancer mortality between 2017 and 2060 by risk category and age under scenarios (A) continued high diagnostic activity as in Stockholm county during 2010 and (B) low diagnostic activity as in Stockholm 1996. Models estimated on all data [file SIM-40-1172-s007.pdf]

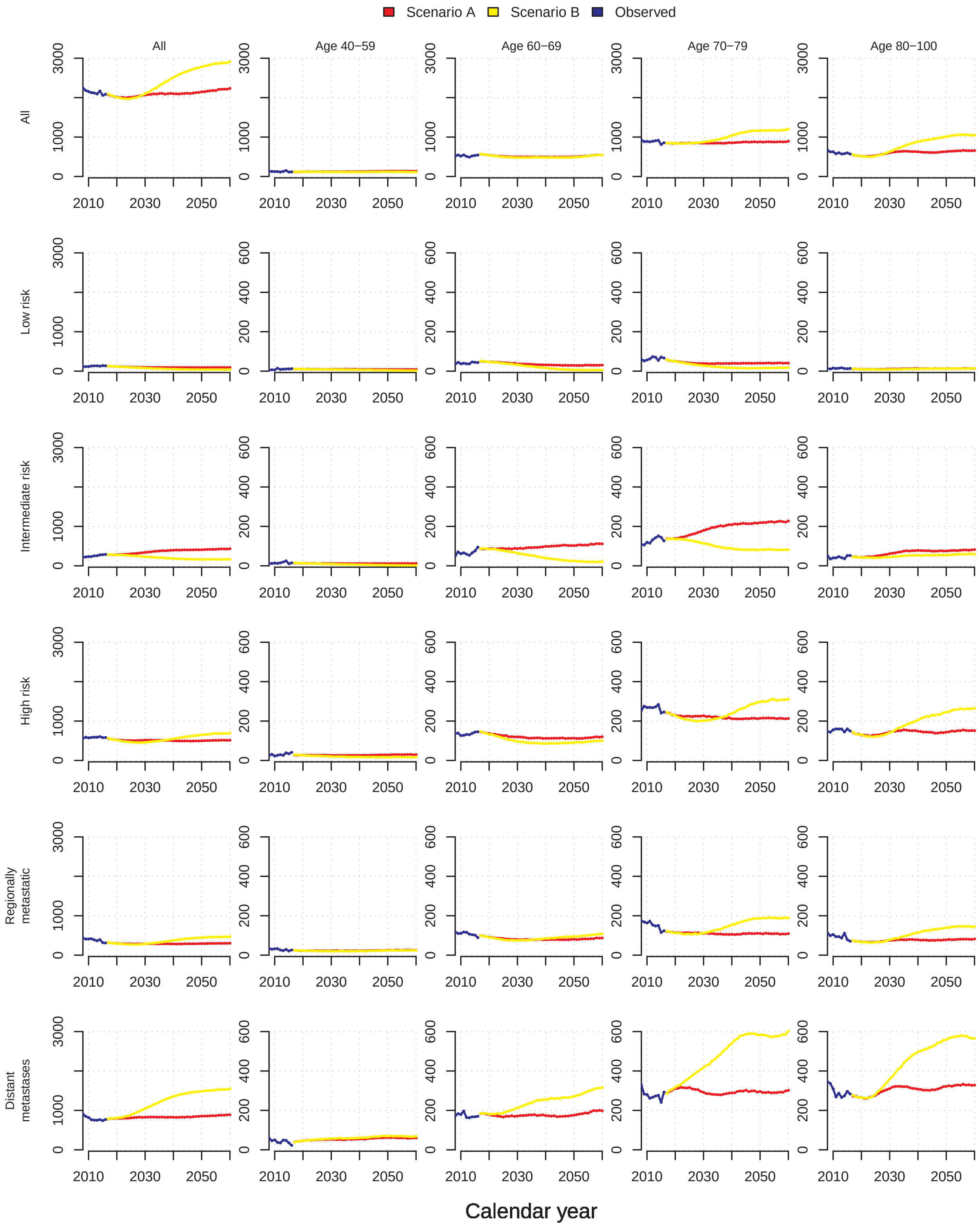

Supplementary Figure 7. Simulated prostate cancer mortality between 2017 and 2060 by risk category and age under scenarios (A) continued high diagnostic activity as in Stockholm county during 2010 and (B) low diagnostic activity as in Stockholm 1996. Models estimated on all data.
